# Register-based study concerning the problematic situation of using interpreting service in a region in Sweden

**DOI:** 10.1186/s12913-019-4619-7

**Published:** 2019-10-22

**Authors:** Emina Hadziabdic, Katarina Hjelm

**Affiliations:** 1 Department of Health and Caring Sciences, Faculty of Health and Life Sciences, Linnaeus University, SE-351 95 Växjö, Sweden; 2 Department of Public Health and Caring Sciences, Uppsala University, SE- 751 22 Uppsala, Sweden

**Keywords:** Incident reporting, Interpreting service, Registers

## Abstract

**Background:**

Due to increasing international migration, Sweden has become a multicultural and multilingual society, with about 19% of the population born abroad, which imposes high demands on the healthcare sector and interpreting services. The aim was to investigate problems in the use of interpreters as recorded by healthcare staff and the interpreter service in a region in Sweden.

**Methods:**

Cross-sectional register-based study. The study focused on a geographically well-defined region in Sweden including (a) specialized care at three hospitals; (b) local healthcare, including out-patient clinics at hospital and emergency healthcare and primary healthcare; and (c) dental care. The study was based on 726 existing incident reports on the interpreting service and information from the interpreter agency from 2012 and the first quarter of 2016 during a period of a massive influx of refugees.

**Results:**

The highest number of adverse advents was reported in local healthcare and mainly concerned the absence of an interpreter at the appointed time. Non-authorized in-person interpreters performed most interpretation assignments and Arabic was the most requested language.

**Conclusions:**

This study highlights the significance of good cooperation between healthcare and the interpreter service in order to guarantee safe and high-quality healthcare for patients in need of interpreters to be able to communicate in healthcare.

## Background

With increasing international migration, from about 150 million migrants 10 years ago to 241 million in 2017, migrants’ health has become a central public health concern [1]. Due to the extensive international migration about 19% of the Swedish population were born abroad and the migrant population is a very heterogeneous group including persons from countries ranging from Finland to Afghanistan [2]. In 2015 an unusually high number of migrants from Afghanistan, North Africa and Syria applied for asylum to nearly all European countries. The third largest recipient country was Sweden [3]. Many immigrants have poor self-perceived general health and psychological well-being [4, 5] related to the migratory process, with increased stress as a result of adaptation to a new life in the host country [6], and asylum seekers seem to utilize health services at a higher rate than the host population, but they face many barriers to care due to communication difficulties [7].

Communication is important in healthcare because of the impact communication has on the delivery of person-centred and safe, high-quality healthcare [8, 9]. Several studies have shown how communication barriers or different cultural backgrounds increase the risks to patient safety [10] and the possibility of misunderstandings concerning the information in hospital discharge instructions [11]. Previous systematic reviews [12, 13] have shown that the use of professional interpreters reduced communication errors and increased patient satisfaction with communication, and the length of hospital stay was shorter [14] . Previous studies have found that [15, 16] interpretation performed by ad hoc interpreters (nurses and social workers) was significantly more likely to have potential clinical consequences in comparison with the use of professional interpreters. Patients and health professionals are generally positive to professional interpreters, but the degree of accessibility varies and is often limited in relation to the need [17–22]. Further, problems in different contexts in healthcare were described, as the interpreters provided by the interpreting service did not match the requirements, e.g. the right language, resulting in a lack of available professional interpreters in a particular language, misunderstandings, the absence of a professional interpreter at the agreed time, limited access to an interpreter agency, and limited access to interpreters of appropriate gender in relation to those in need of interpreters [23, 24]. Our previous qualitative study on incident reports concerning the use of interpreters in a particular primary healthcare centre [24] found that the content of incident reports mainly focused on problems related to the interpreters’ language competence and organizational issues concerning interpreter services.

Worldwide, various policies and laws have been developed to improve patient safety through advocacy, collaboration and partnership [8]. The purpose of incident reports is to function as a patient safety reporting system. Further, incident reports are planned to produce a clear, valuable response to validate the resources expended and to ensure learning from others’ experience in order to use the results to formulate and disseminate recommendations for system change [25]. In Sweden, all healthcare staff are obliged to be involved in systematic work on quality improvement [26] and to give patients an opportunity to take part in patient safety work according to the Patient Safety Act (SFS 2010:659) as a part of systematic quality work [26]. Existing incident reports written by healthcare professionals are studied by the head of the department, who in teamwork with healthcare staff analyses the content and decides on procedures to be taken to avoid any repetition of the incident and to develop methods in the organization in order to increase the quality of the work [25, 26].

However, a previous study [27] has found that incident reports from healthcare staff are under-represented especially when it comes to reporting adverse events to a superior. Therefore, this study offers a unique opportunity to analyse which problems were recorded by healthcare professionals and interpreter service concerning the use of interpreters during the period from 2012 to the first quarter of 2016. These years were a period of a massive influx of migrants with different foreign languages and a particularly high demand for interpreters in healthcare, which the health service and interpreting service was not prepared for, and the question is how the organization can be improved and developed in order to offer information about where it fails, thus making it possible to improve clinical practice [25].

### Aim

The purpose of this study was to investigate problems concerning the use of interpreters as reported by healthcare professionals and the interpreter service in a region in Sweden.

## Methods

### Design

A cross-sectional register-based study design using professional interpreters from a county council was used to analyse the influence on healthcare in 2012–2016 (years with the highest ever number of refugees) [28].

### Setting

The study focused on a geographically well-defined region in Sweden with approximately 454,307 inhabitants [29]. The region was formed on 1 January 2015 through a reorganization of a county council. The healthcare system in the region involved: (a) specialized care delivered at three hospitals in the region (including a regional hospital and two county hospitals); (b) local healthcare including out-patient clinics at hospital and emergency healthcare and primary healthcare including healthcare centres; and (c) dental care services including general, specialist and hospital dental care.

Healthcare in the region is governed by Swedish legislation, including five different laws: 1) the Health and Medical Services Act (SFS 2017:30), 2) the Patient Act (SFS 2014:821), 3) the Public Procurement Act (SFS 2007:1091), 4) the Management Act (SFS 1986:223) and 5) the Dental Care Act (SFS 1985:125). The main aim in healthcare and dental care is delivery of high-quality care with good accessibility, where individuals should be treated equally and according to their individual needs (Health and Medical Services Act 2017:30, Dental Care Act 1985:125). Patients have the right to receive information about the state of their health and treatment and the information should be adapted to the individual’s conditions as regards age, maturity, experience and linguistic background (Patient Act 2014:821). According to the Management Act (SFS 1986:223) interpreters should be used in all contacts with public authorities for persons who cannot speak Swedish, and the responsibility for calling upon the interpreter service and obtaining the provision of an interpreter lies with the healthcare service. The Public Procurement Act (SFS 2007:1091) stipulates that contracts, in this case with the interpreter service, must be signed with the most economically beneficial interpreter agency after an assessment based on criteria of price and quality. Further, institutions in the region follow the rules of the National Board of Health and Welfare on management systems for quality and patient safety in healthcare [30].

There are two types of interpreters employed by the Swedish interpreter agencies: (a) authorized interpreters with university training, who undertake special tests performed by the Administrative Services Agency and acquire special skills as a medical interpreter or a court interpreter; and (b) non-authorized interpreters with basic training for interpreters or no training at all, employed at the interpreter agencies [31].

### Procedure and data collection

After a seminar, presenting data from another study by the authors, an emergency coordinator at the county council offered the research group data collected for the planning of healthcare to meet the needs of refugees. The head manager and a lawyer from the county council were contacted to obtain approval for the study. Copies of registers on problematic experiences of using interpreters in a county council, in the form of incident reports, were handed over to the authors after anonymization. Incident reports should be written by healthcare staff as a part of systematic quality work [26].

The data register included 726 incident reports on the use of interpreters employed by the interpreter agencies, written by different professions in the healthcare service from 2012 to the first quarter of 2016, and information from the interpreter service.

### Analysis

Bivariate descriptive statistical analysis was performed using correlation procedure to describe correlations between two variables [28]. Pearson was used for ratio measures and Spearman for ordinal measures. Further, descriptive statistics (frequencies and percentage) were used. Data were systematically organized in numeric values from highest to lowest, together with a count and percentage of the number of times each value was obtained [28].

## Results

During the year 2015, there were 7009 registered requested consultations with interpreters and among these 194 registered incident reports.

The results showed that the frequency of incidents reported regarding interpretation did not differ between care in different institutional contexts (*p* = 0.059), but there was a tendency to a weak association between care in different institutional contexts (specialized care, local healthcare and dental care) and the year of reporting 2012–2015 (*p* = 0.02, Spearman rho = 0.138).

The descriptive analysis of data registers on experiences of using interpreters by a county council from 2012 to the first quarter of 2016 showed that the highest number of incidents was reported in 2015 (see Table 1) in local healthcare (see Table 2), and the reason for the adverse incidents mainly concerned the absence of an interpreter at the appointed time (see Tables 3 and 4) in 2013 (see Table 5). Further, the assignments were mostly performed by non-authorized in-person interpreters (see Table 6) during working hours from Monday to Friday, 7 a.m. to 7 p.m. (see Table 7) and Arabic was the most frequently requested interpreter language in 2015 (see Table 8).

**Table 1:** Incident reports reported between 2012 and the first quarter of 2016

Year	Number of incident reports	Percentages
2015	194	22
2014	191	26
2013	169	23
2012	153	21
first quarter of 2016	18	3
Missing	1

**Table 2:** Incident reports reported in different areas of healthcare

Area of healthcare	Number of incidents report	Percentages
Local healthcare	451	61
1) Primary care	254	35
2) Refugee medical Center	129	17.8
3)Out-patient clinics at hospital	57	7.9
4) Emergency care	2	0.3
Dental care	140	19.3
Specialized care	142	19.6
External care providers	2	0.3

**Table 3:** Reasons for reporting the incident reports

Reason	Number of incident reports	Percentages
Absence of interpreter at appointed time	213	29
Deviation regarding interpreter agency	160	22
Telephone interpreter does not answer	96	13
Interpreter delayed	88	12
Limited interpreter competence	70	10
Other complaints	67	9
Disturbing sounds during telephone interpreting	20	3
Misunderstanding	12	2

**Table 4:** Data concerning the distribution of reasons for reporting the incident in the respective healthcare context

		Reasons for reporting the incidents							
Healthcare context	Mis-understanding	Absence of interpreter at appointed time	Other complaints	Interpreter delayed	Deviation regarding interpreter agency	Limited interpreter competence	Telephone interpreter did not answer	Disturbing sounds during telephone interpreting	Total
Primary healthcare	3	53	29	34	68	21	40	6	254
Refugee medical centre	1	46	4	9	35	10	20	4	129
Out-patient clinics at hospital	1	17	8	6	6	13	5	1	57
Emergency care	0	0	0	0	1	0	1	0	2
Dental care	3	53	12	23	17	14	11	7	140
Specialized care	4	43	14	16	32	12	19	2	142
External care providers	0	1	0	0	1	0	0	0	2
Total	12	213	67	88	160	70	96	20	726

**Table 5:** Data concerning the distribution of reasons for reporting incidents in each year

Reasons for reporting the incident reports	Year					Total
	2012	2013	2014	2015	2016	
Misunderstanding	3	2	4	2	0	11
Absence of interpreter at appointed time	51	70	41	48	3	213
Other complaints	15	15	14	21	2	67
Interpreter delayed	22	19	27	16	4	88
Deviation regarding interpreter agency	37	28	53	40	2	160
Limited interpreter competence	10	14	19	23	4	70
Telephone interpreter did not answer	13	17	28	35	3	96
Disturbing sounds during telephone interpreting	2	4	5	9	0	20
Total	153	169	191	194	18	725

**Table 6:** Number of interpretations performed during 2015

Type of interpreters	Authorized interpreters within healthcare		Other authorized interpreters		Non-authorized interpreters	
Form of interpretation	Interpreter on spot	Telephone interpretation	Interpreter on spot	Telephone interpretation	Telephone interpretation	Interpreter on spot
Number of interpretations performed	4	9	167	210	4868	21,577
Percentages of interpretation performed	0.02	0.03	0.62	0.78	18.14	80.41
Total number of interpretations performed	26,835

**Table 7:** Data concerning distribution of assignments during the day in 2015

	Number of assignments	Percentages of assignments	Number of hours	Percentage of hours
Weekdays 7 a.m.–7 p.m.	25,856	97	29,677.9	95
Weekdays 7 p.m.–7 a.m.	623	2	794.6	3
Weekends	384	1	735.4	2

**Table 8:** Data concerning the top languages requested in 2015

Language	Number of request	Percentages
Arabic	2785	39.7
Somali	1553	22.2
Other small languages	1017	14.5
Tigrinyan	537	7.7
Dari	354	5.1
Persian	281	4.0
Bosnian/Serbian/Serbo-Croatian	210	3.0
Albanian	115	1.6
Sorani	87	1.2
Polish	70	1
Total	7009	100

## Discussion

The study’s most notable finding was that that the highest number of incidents during the period from 2012 to the first quarter of 2016 was reported in local healthcare and the reason for the adverse incidents mainly concerned the absence of an interpreter at the agreed time. Also, 80% of interpretation assignments were mostly performed by non-authorized in-person interpreters and Arabic was the most requested interpreter language in 2015.

The high number of incident reports showed that healthcare professionals were aware of the influence that communication has on the delivery of person-centred, safe and high-quality healthcare. It has previously been found [32] that healthcare staff who work in an organizational culture that inspires reporting and avoids guilt considered that this kind of organization led to the improvement of patient safety and communication between healthcare staff and patients. This study indicates the need for structures in the organization that support healthcare professionals to write incidents reports concerning failure in the interpreter service as a part of systematic quality work [26], because communication is considered as an important area for the improvement of patient involvement in matters of patient safety [33]. Challenges in communication through interpreters have been described as being related to interpreters’ personal characteristics such as linguistic competence [21, 34, 35] and professional attitude and to the organizational context of interpreting practice, including the availability of laws, policy and guidelines [21, 35]. Failure in interpreting practice may lead to inadequate communication, which in turn may cause delayed care [15], limited access to diagnoses, diagnostic testing and treatment [36]. Thus, despite the evidence that staff participation in incident reporting [27, 37] and use of trained interpreters [38–40] is often suboptimal; the results of this study indicate that the problem is even greater in reality. The present study supports the importance of learning from incident reports in order to reduce barriers to care for patients who are in need of interpreters to be able to communicate in the healthcare service.

The study found that the highest number of incidents was reported in local healthcare in the area of primary healthcare, in contrast to specialized care where fewer problems were found. Primary and specialized are two dissimilar contexts that vary in both the aims and the character of the care practices they deliver. In primary healthcare consultations are generally highly structured and delivered in a limited time frame, in contrast to specialized care where the care is delivered in both a limited timeframe and a limited space. A previous study [35] showed that the use of professional interpreters was related to the organizational context where professional interpreters were not used in ambulance service; instead those available at the time, e.g. relatives and bilingual staff, were used to a greater extent. Migrants have poorer health status on account of the migratory process, with increased stress as a result of adaptation to a new life in the host country [6]. Thus, migrants can be vulnerable [41] due to global differences in disease epidemiology, lifestyle, culture and discrimination [42] and they have a tendency to use more healthcare than native-born populations do [7, 43] and thus, primary healthcare is often the first point of entry to the healthcare system in most countries [44]. The characteristics of the environment and context in which care is delivered need to be taken into consideration in order to provide a suitable and effective interpreting service in local healthcare to meet the common and everyday needs of people.

Findings in this study showed that a large majority of the adverse incidents described dissatisfaction due the absence of an interpreter at the appointed time. This finding may be understood as occurring because of interpreters’ employment conditions in what is an unregulated business from the central level of the organization [31]. The interpreter agency organizes interpreters’ work and schedules, and the interpreters receive payment on a time-unit basis. Thus, interpreters work as freelancers for one or more interpreting agency and this could negatively influence their performance and the quality of the interpreting [45]. In order to address this problem, there is a need for better organization of interpreters’ employment conditions, and the interpreter service needs to be integrated in the organizational routines of healthcare.

This study found that non-authorized in-person interpreters mostly performed interpretation. In Swedish healthcare, there is a lack of authorized interpreters, and this trend is projected to remain because (a) the training that is available to interpreters is not sufficient to meet the needs of the healthcare service, and (b) the healthcare sector offers lower payment than others in the public sector [31]. Further, this study showed that 87% of telephone interpreters did not answer at the booked time. A previous study [45] found that interpreters whose competence and interpreting skill are lower were common on the agencies’ telephone lists and are employed by agencies who only invest small sums in authorized interpreters and training; these are the agencies that win contracts with healthcare due to the current situation which considers the price more than quality as a result of the Public Procurement Act (SFS 2007:1091). It is important to be aware of this because it can negatively affect communication, patient safety and the quality of healthcare. In order to resolve this problem, there must be a will to have central control over interpreter services, and thus interpreter agencies. As it stands today, the interpreter service has largely developed with no control at the central level [31].

### Limitations

A potential problem with the chosen data collection method could be the absence of background data characteristics relating to those who wrote the records [28]. At the same time, the registered data were useful in this study for giving an opportunity to investigate what was reported by healthcare and the interpreter service concerning the use of interpreters, and to cover a period of massive influx of migrants with foreign languages when the need for interpreters increased significantly, which the healthcare organization was not prepared for. Thus, the registered data gave a homogeneous picture of the problems, and the data obtained in this study offer a deeper understanding of the area investigated. However, it would need a further study with a larger sample, also including other county councils, to be make it possible to generalize the results.

Another potential limitation of the study could be that the researchers had no control over how the number of instances of interpreter use was calculated/documented. However, considering the evidence that both staff participation in incident reporting is under-reported [27, 37] and use of trained interpreters is often underused [38–40]: this may indicate that in reality the problem is even greater.

## Conclusion

This investigation, based on existing data registers concerning problems related to use of interpreters employed by the interpreter agencies, showed that the highest number of adverse advents was reported in local healthcare and the main reason for the adverse incidents was the absence of an interpreter at the agreed time. The interpretation was mostly performed by non-authorized in-person Arabic-speaking interpreters.

### Clinical implication

In our increasingly heterogeneous societies, the findings from this study help to fill a knowledge gap concerning the development of cooperation between healthcare service and the interpreter service in order to reduce barriers to patient safety and to improve the quality of healthcare for patients who are in need of interpreters to communicate. However, the results indicated the need of further investigation with a larger sample especially of the evidence that problems related to the use of interpreters are under-reported.

Further, on a national level it is important to guarantee good accessibility to authorized interpreters with regulated employment conditions, in consideration of the unpredictable and urgent requirements for interpreters in healthcare.

## Data Availability

In order to protect integrity, anonymity and confidentiality of the respondents, data will not be shared.

## Abbreviations

a.m.: Ante meridiem: Before noon
p: *P*-values
p.m.: Post meridiem: After noon
Spearman rho: Spearman’s rank correlation coefficient
